# Multisonic Ultracleaning and Laser-Activated Irrigation Effect Compared to Passive Ultrasonic Activation for Debridement in Minimally Invasive Instrumentation of Necrotic Oval Root Canals: An Ex Vivo Histological Analysis

**DOI:** 10.3390/jcm14082597

**Published:** 2025-04-10

**Authors:** Mustafa Gündoğar, Olcay Özdemir, Özgecan Gündoğar, Sibel Bektaş, Fadile Nur Demir, Nergiz Bolat

**Affiliations:** 1 Department of Endodontics, Faculty of Dentistry, Istanbul Medipol University, 34815 Istanbul, Türkiye; 2 Department of Endodontics, Faculty of Dentistry, Karabük University, 78050 Karabük, Türkiye; 3 Department of Pathology, Gaziosmanpaşa Training and Research Hospital, University of Health Science, 34255 Istanbul, Türkiye; ozgecankarahan@hotmail.com (Ö.G.); sibel_bektas@yahoo.com (S.B.); fadilenur@gmail.com (F.N.D.); 4 Department of Endodontics, Faculty of Dentistry, Suleyman Demirel University, 32260 Isparta, Türkiye; nergizbolat@gmail.com

**Keywords:** GentleWave system, histological analysis, irrigation activation, laser-activated irrigation, passive ultrasonic activation, root canal debridement

## Abstract

**Objectives**: This study aims to evaluate the effectiveness of current conventional and advanced irrigation techniques after minimally invasive instrumentation in necrotic oval root canals by histological analysis. **Methods**: Seventy extracted necrotic lower premolars with single oval canals classified utilizing bidirectional radiographs (mesiodistal diameter 2.5 times larger than buccolingual) were prepared up to 20.04 v. The samples were assigned to five experimental groups (n = 14) using the complementary irrigation technique: needle (control), passive ultrasonic, and shockwave-enhanced emission photo-acoustic streaming activation using Er:YAG laser (SWEEPS), Er,Cr:YSGG laser (Waterlase iPlus), and multisonic ultracleaning technology (GentleWave). After irrigation protocols, the roots were demineralized and the apical 5 mm was multi-sliced and processed for histologic examination. The residual necrotic tissue and debris percentage was calculated via image analysis software. One-way ANOVA and Tukey’s test were used to verify the variables influencing debridement (*p* < 0.05). **Results**: The mean value of the GentleWave group was the record low at 1.54 ± 1.46, and the utmost was needle irrigation with 15.64 ± 7.23. The main effect of techniques on the debridement was statistically significant (*p* < 0.001). The course of debridement effectiveness, according to the levels of significance between the groups, was as follows: Multisonic ultracleaning > Er:YAG > Er,Cr:YSGG > Passive ultrasonic irrigation > Needle irrigation (*p* <0.05). **Conclusions**: In necrotic oval-shaped canals after minimally invasive instrumentation, multisonic ultracleaning with updated software was considerably more effective in removing remnants in the apical level. Er:YAG and Er,Cr:YSGG lasers were highly promising, with results close to multisonic ultracleaning. It should be considered that needle irrigation and passive ultrasonic activation may not be able to provide competent debridement in treating such types of root canals.

## 1. Introduction

The development of materials and device technology, enhanced nickel–titanium tools, advanced irrigation protocols, magnification systems, and a better understanding of the biological behavior of the root canal system and pathogenicity have provided the ability to manage complex endodontic cases and associated symptoms at a single visit [1]. Accurate shaping and cleaning of the whole system of root canals have been a leading challenge, particularly in narrow, curved, or oval-shaped canals and anatomical complexities [2,3]. Considering the resistant biofilm layer containing various microorganism clusters and the complicated anatomical structure of the root canal system, it is recommended to increase the effectiveness of solutions by activation methods to disinfect the root canal system and remove residual tissues by debridement [4,5,6].

For this purpose, the passive ultrasonic method, widely used in daily clinical practice, is accessible and low-cost. It is based on the principle of activating the movement of solutions with ultrasonic waves. In addition, the irrigation activation efficiencies of laser systems have been proven by in vitro experiments [7]. Shockwave-enhanced emission photo-acoustic streaming (SWEEPS, Skypulse Laser, Fotona, Ljubliana, Slovenia) is a new mode of Er:YAG laser used for irrigation activation [7,8]. The mechanism provides the formation of shockwaves and turbulent fluid movement. As a result, the effectiveness of the debridement can be increased significantly [8]. Er, Cr: YSGG (Waterlase iPlus, BiolaseTech-nology Inc., San Clemente, CA, USA), is a laser system used for the cleaning, disinfection, and debridement of the root canal system [9]. The system has a different approach and promotes intracanal laser operation with a high efficacy in endodontics due to the high affinity that erbium lasers have for water, which justifies its use as irrigation activation. The wavelengths of Er:YAG and Er,Cr:YSGG lasers are different (2940 vs. 2780 nm), and the effects on energy absorption and heat creation are considerable [10,11,12].

The multisonic ultracleaning system (GentleWave^®^, Sonendo Inc., Laguna Hills, CA, USA) has recently come to the forefront in canal cleaning efficiency as a device that can generate a broad soundwave spectrum and create advanced fluid dynamics [13,14]. Recently, the company introduced a developed system for GentleWave^®^ as G4 System with ProControl^™^ software update to enable NaOCl concentration adjustments up to 5% for vital tissue cases and optimized debridement of anatomical complexities [15].

Since root canal shaping procedures reduce the mechanical resistance of tooth hard structures [16,17], it is essential to perform a high-level debridement with solution activation with the least possible preparation [18,19,20,21]. According to the accessible evidence in the literature, the available data are quite limited when the debridement efficiency of root canals is evaluated histologically after a minimally invasive preparation. In addition, no comparative evidence regarding histological debridement evaluation has been found for the most up-to-date irrigation activation methods, such as Er:YAG, Er,Cr:YSGG laser systems, and multi sonic ultracleaning irrigation, compared to passive ultrasonic activation in necrotic oval root canals with minimally invasive instrumentation.

In this context, considering the limitations of roots canal debridement with minimally invasive instrumentation regarding residual necrotic tissue and multimicrobial biofilm remnants and lack of evidence of which protocol may have potential as a gold standard for root canal cleaning effectively after limited mechanical preparation, this study aimed to evaluate the effectiveness of current advanced techniques used for irrigation activation such as Er:YAG, Er,Cr:YSGG laser systems, and multisonic ultracleaning irrigation compared to passive ultrasonic activation and conventional needle irrigation after minimally invasive preparation in necrotic oval root canals by histological analysis. Therefore, the methodology followed for the study included several key steps to ensure the usability of minimally invasive preparation techniques and which irrigation activation technique provides more effective debridement in necrotic oval root canals. The null hypothesis of the study was that there was no difference between the activation methods applied in root canals shaped with minimally invasive preparation.

## 2. Materials and Methods

Ethical approval was obtained from the local Non-Interventional Ethics Committee (protocol no: 2024/2157). The details of the applied methodology are outlined below: sample size calculation, sample selection, and eligibility; preparation of root canals; allocation of groups and root canal debridement; histological analysis and morphometric evaluation; and statistical analysis. This comprehensive methodology ensures a thorough assessment of the effectiveness of current advanced techniques used for the activation of irrigation solutions such as Er:YAG laser (SWEEPS), Er,Cr:YSGG laser (Water-lase iPlus), and multisonic ultracleaning technology (GentleWave) compared to passive ultrasonic irrigation activation with needle irrigation as a control during root canal treatment after minimal invasive instrumentation in necrotic oval root canals by histological analysis.

### 2.1. Sample Size Calculation, Sample Selection, and Eligibility

The sample size was calculated via G*Power software (V3, Kiel University, Kiel, Germany) using a difference between independent means with a power of 0.77, an alpha error of 0.05 and 1-beta error of 0.95. Based on an analysis report from a previous study [22], the number for each group was determined as 14 using a total of 70 teeth for the study.

The inclusion criteria were necrotic oval lower premolar teeth with a single root canal in the best possible standardization, extracted from patients aged 18–35 that had teeth that indicated pulp necrosis supported by radiography or vitality tests with unfavorable prognosis considering large periapical pathosis with/without periodontal loss or post-endodontic restorability. Although no tooth extraction was performed specifically for the study, regarding ethical considerations, samples were selected from extracted teeth that met the inclusion criteria through written informed consent of the patients for usability. The exclusion criteria were multiple root canals, not oval structures, structurally different, immature, endodontically treated before, and cracks or fracture lines.

Determination of the oval root canal configuration was conducted as follows: measurement of the space corresponding to the root canal lumen 5 mm from the apex. The root canals were classified as oval when the mesiodistal diameter was 2.5 times larger than the buccolingual diameter with periapical radiography [2]. The included samples were labeled 1 to 70.

### 2.2. Preparation of Root Canals

After a sufficiently wide but minimal access cavity preparation, patency was per-formed using a 10 K file (M-Access, Dentsply Maillefer, Ballaigues, Switzerland) for standardization. The working length was recorded as 1 mm behind the file after the file was seen from the apex. The lengths of the samples were equated after the working length measurement by occlusal reduction. Afterward, the samples were embedded in a silicone impression material (C type, Zhermack Zetaplus, Badia Polesine, Italy) to simulate root sockets and a closed canal system.

The TruNatomy file system (Dentsply Sirona Endodontics, Ballaigues, Switzerland) was used as follows: Glider and Prime (20.04 v), respectively, according to the manufacturers’ recommendation (500 rpm/1.5 Ncm) for minimal invasive preparation. A measure of 2 mL sodium hypochlorite (5%, Microvem AF, Altun Medical, Sakarya, Türkiye) was used between file changing and administered by a needle with 30 G tip (Irrigation Needle™, Dentsply Sirona Endodontics, Ballaigues, Switzerland).

### 2.3. Allocation of Groups and Root Canal Debridement

The 5 groups of 14 unique numbers per set in the range from 1 to 70 were allocated using https://www.randomizer.org software for the double-anonymized experiment (accessed on 10 June 2024).

- **Needle Irrigation (NI) (n:14):** The root canals were irrigated via needle tip without activation in the same preparation using 3 mL of sodium hypochlorite, 2 mL of 17% EDTA (Imicryl, Imicryl Dental, Konya, Türkiye), and 3 mL of 5% sodium hypochlorite, respectively.
- **Passive Ultrasonic Activation (PUI) (n:14):** An ultrasonic tip (#20) at 30 kHz frequency was used to activate the solution in the root canal system. The root canals were irrigated with solutions, and the tip was placed 1 mm short of the working length. Three turns of cycles per solution using 3 mL sodium hypochlorite, 2 mL of 17% EDTA, and 3 mL of 5% sodium hypochlorite were repeated with refreshment of the solutions for 20 s, resting the solutions for 20 s inside the canal, and activation for 20 s with around 1–4 mm back-and-forth movement, respectively. All solutions were activated by the mentioned tool and protocol.
- **Er:YAG (SWEEPS) (n:14):** A Skypulse laser with an air–water cooling-off application was used to activate the solution in the access cavity. Three turns of cycles per solution using 3 mL sodium hypochlorite, 2 mL of 17% EDTA, and 3 mL of 5% sodium hypochlorite were repeated with refreshment of the solutions for 20 s, resting the solutions for 20 s inside the canal, and activation for 20 s, respectively. All solutions were activated by the aforementioned tool and protocol.
- **Er,Cr: YSGG (n:14):** A Waterlase iPlus laser was used to activate the solution in the root canal system with a fiber tip (RFT2-25). The console was set to 50 Hz, 25 mJ, and 1.25 W with an air–water cooling-off application. The tip was placed 1 mm short of the working length. Three turns of cycles per solution using 3 mL sodium hypochlorite, 2 mL of 17% EDTA, and 3 mL of 5% sodium hypochlorite were repeated with refreshment of the solutions for 20 s, resting the solutions for 20 s inside the canal, and activation for 20 s with around 1–4 mm back-and-forth movement, respectively. All solutions were activated by the aforementioned tool and protocol.
- **Multisonic ultracleaning system (n:14):** A GentleWave^®^ G4 with ProControl^™^ software system with a CleanFlow handpiece was used for activation. The handpiece was connected to the main console following the manufacturer’s recommendations. An occlusal platform was fabricated to adapt the SoundSeal (Sonendo Inc., Laguna Hills, CA, USA) handpiece to the airtight seal access cavity. The mechanism of fluid dynamics was based on the action generated by the GentleWave console, which was set and operated. Measures of 5% sodium hypochlorite, 17% EDTA, and 5% sodium hypochlorite were used with distilled water in between for 5 min at a flow rate of 45–50 mL/min.

### 2.4. Histological Analysis and Morphometric Evaluation

All the root canals were flushed with 1 mL of distilled water and dried to proceed with the histological analysis. The samples were immersed in buffered formalin (10%, 48 h) and demineralized in formic acid solution (22.5%, *v*/*v*) and sodium citrate solution (10%, *w*/*v*) for 3 weeks. The endpoint was monitored using radiography. The samples were rinsed in tap water (24 h) before being dehydrated and processed for histological analysis. Root samples were embedded in paraffin wax and serial cross-sectional plates (0.3 µm) were obtained from every 0.3 mm from the apical 5 mm levels. As a result, a total of 14 slides were prepared per root. Three optimal cross-sections were selected from the similar 1–3 mm apical levels. The plates were mounted on glass slabs and stained with hematoxylin–eosin.

The slabs were visualized under a light microscope at ×10 magnification (Olympus BX51, Tokyo, Japan). The images were transferred to imaging software (ImageJ software web version, available at https://ij.imjoy.io, accessed on 10 June 2024) to measure the cross-sectional areas of each root canal and remaining residues (mm^2^). The percentages of residual area were calculated for each section. Two independent researchers pre-calibrated for the residues which areas should be considered and, for the area calculation technique, evaluated and calculated all sections for the root canal; mean values were recorded as the section scores.

### 2.5. Statistical Analysis

Statistical analysis was conducted with IBM SPSS (SPSS V23; IBM Corp., Armonk, NY, USA). The normality of the data was evaluated with the Shapiro–Wilk test. One-way ANOVA followed by Tukey’s post hoc test for multiple comparisons were used to compare the percentage of residual tissue for the different irrigation activation techniques. The significance level was taken as 0.050.

## 3. Results

In this study, which aimed to evaluate the effectiveness of current advanced techniques compared to conventional ones, the mean value of the GentleWave group was the record low with 1.54 ± 1.46, and the utmost was needle irrigation with 15.64 ± 7.23. The main effect of the activation techniques on the debridement of root canals was statistically significant (*p* < 0.001) (Table 1 and Table 2).

**Figure 1:**
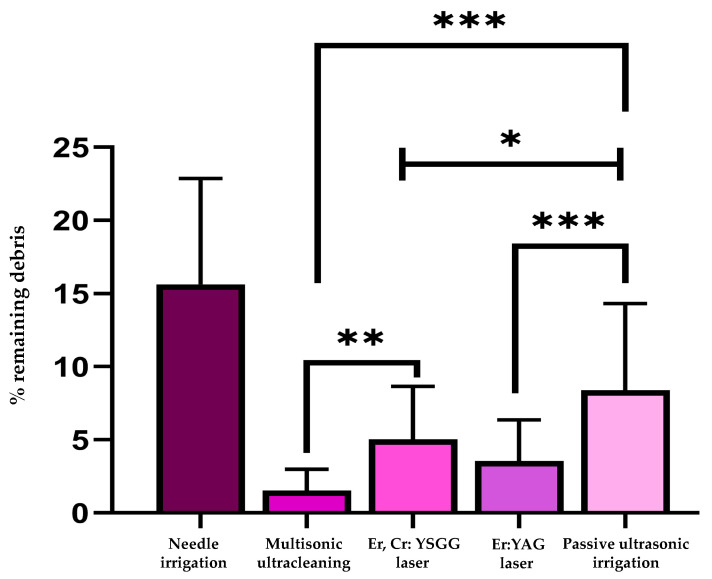
Mean values of the remaining area with significance between groups. Statistical difference information indicated using *, **, *** is given in Table 2.

**Figure 2:**
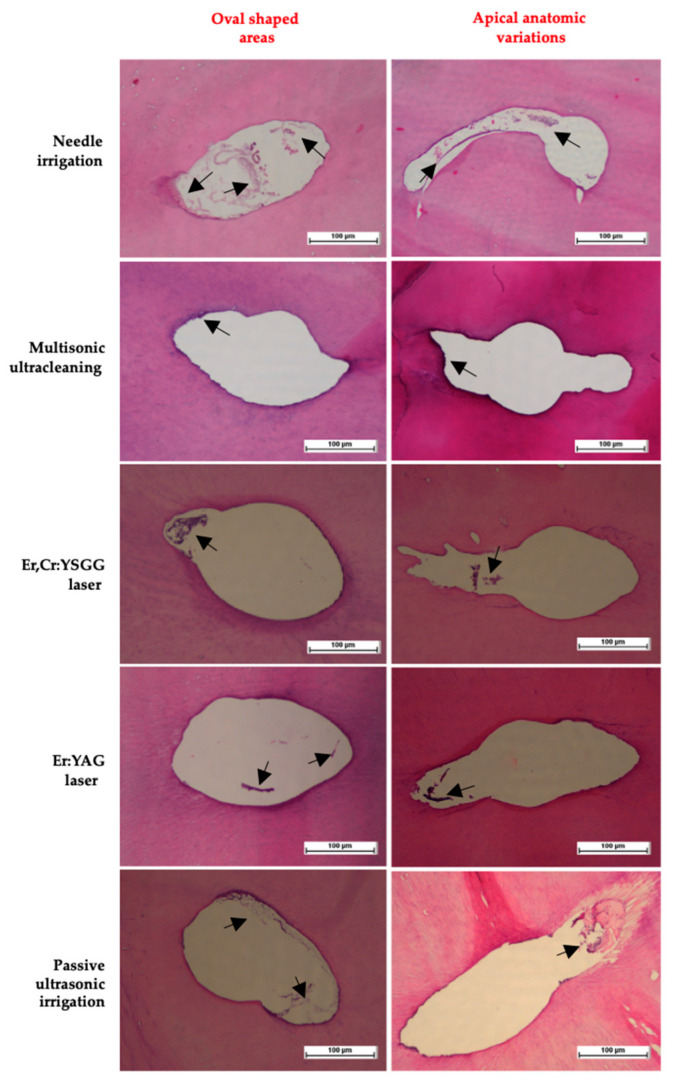
Representative histological analysis staining sections of activation techniques used in this study. Black arrows indicated remaining residues.

The course of debridement effectiveness according to the levels of significance between the groups was as follows: Multisonic ultracleaning > Er:YAG > Er,Cr: YSGG > Passive ultrasonic irrigation > Needle irrigation (*p* < 0.05). Figure 1 shows the mean values of the debris and necrotic tissues’ remaining area values with significance between groups. Figure 2 presents the representative staining sections of the histological analysis of activation techniques used in this study. The existing remnants are marked with black arrows in Figure 2. Especially for the needle irrigation group, the tissue and debris residues were highly significant, as pointed out in the representative histological sections.

## 4. Discussion

The present study investigated the effectiveness of debridement after using five different irrigation protocols in the oval necrotic root canal system of extracted mandibular premolars. Histological analysis was conducted to evaluate the residual reduction. An advantage of the histological approach used in this study is that it is a well-validated method [2,22,23].

Various cavity designs, minimally invasive approaches, and coronal restorative applications to protect an endodontically treated tooth from mechanical distortions are recommended to sustain the long-term prognosis [19,24]. Nowadays, conservative systems are available with a philosophy of minimally invasive endodontics based on fraying out the dentin as minimally as possible to avoid weakened pericervical dentin [20]. Another factor to consider in structural integrity is the taper and apical diameter of the tools used [25]. It is known that instruments with a greater taper lead to higher wear of dentin structure [25,26,27]. Additionally, some authors prefer a conservative approach, mainly when irrigation activation methods are used [23,28,29].

Considering the current clinical conditions with the most conservative perspective possible, this study aimed to assess the debridement efficiency of current and advanced irrigation activation methods, with the null hypothesis of no difference between the products and techniques. Based on the study results, the null hypothesis was rejected because there were techniques that stood out statistically among the products.

Root canal anatomy variations undoubtedly present a challenge to management of endodontic treatment, including shaping and cleaning root canals [22]. Considering the design of engine-driven files, the instrumentation is intricate, especially in oval canals, as untouched areas remain in the extensions of the flattened canals. Based on this context, since the main focus of the study was minimally invasive preparation, oval canals were preferred to more effectively evaluate the efficiency of targeted minimal instrumentation and untouched areas debridement. The reason for using mandibular premolar teeth is that the oval canal structure is seen more frequently, in addition to other complexities [3]. Wu et al. reported that instruments can only contact 40% of the apical walls within the oval canals when a rotating technique is used [30]. Therefore, the apical area was preferred to interpret the methods and tools by histologic analysis and morphometric evaluation.

The total solution delivery time and volume are critical for debridement. In daily clinical conditions, the volume of used irrigants can vary from 1 to 15 mL regarding concentration and retention time [31,32]. The main advantage of the multisonic ultracleaning system is that it circulates approximately 45–50 mL of solution in the canal in a short time of a minute [28]. It is essential to standardize all the parameters possible when conducting an ex vivo study, except for the variable that will be the focus of the evaluation. Considering the techniques compared, the concentration of the solutions was approximately standardized; however, attempting to standardize the volume and the mechanism of irrigant activation in both methods is impractical and would not reflect the clinical usage of the techniques. Nevertheless, to avoid this limitation as much as possible, a 10 mL volume of liquid delivery was provided in other systems. Based on the data obtained from this study, the advanced technology multisonic ultracleaning by the GentleWave system provided more effective debridement than all other activation methods. This finding is not surprising considering the total fluid volume circulated during the processing time, even in a short period of 5 min. To the best of our knowledge, there is no study concerning using the updated software of the GentleWave system (GentleWave^®^ G4 System with ProControl^™^). Thus, this study represents the first and only analysis of its kind in the field.

The effectivity of root canal debridement is generally measured by evaluating several parameters, such as antibacterial effect, residual pulp tissue, necrotic tissue, and debris removal. These parameters are considered crucial to declare the overall cleaning quality of a particular irrigation activation technique. In general, the ability of ultrasonic irrigation, compared to needle irrigation, to purify root canals is not a new finding [22,28]. The currently accepted approach to root canal treatment is that even with extensive preparation, needle irrigation is insufficient for effective disinfection and debridement [23,33]. The effectiveness of irrigation activation strategies becomes even more critical when mechanical preparation approaches focus on minimally invasive, especially oval canal systems. In a recent study, Huynh et al. reported that a laser-based irrigation strategy was significantly more effective in removing debris than sonic systems in minimally instrumented root canal systems [9]. When the literature is evaluated in general, the effectiveness of sonic and ultrasonic systems relative to each other is controversial, depending on the canal shape, anatomy, and root region [34,35]. In this study, activation using two different laser systems proved to exhibit significantly superior properties compared to needle irrigation and passive ultrasonic activation, which will contribute to the evidence gap in the literature on how effective Er:YAG laser by SWEEPS and Er,Cr:YSGG laser by Waterlase iPlus may be even in a conservative preparation. Insomuch as the residual area values of multisonic ultracleaning by GentleWave were considered, the used laser systems presented very close results, even at a volume of 10 mL.

The basic action mechanism of laser systems is the succession of very rapidly growing and imploding vapor bubbles within the irrigation solution. Erbium lasers work in pulse mode, so do not emit continuously and deliver short pulses of energy [36]. The superheating of the aqueous liquids and the high absorption capacity of lasers cause the formation of vapor by explosive boiling [37,38]. This situation creates high-speed fluid motion around the expanding and imploding bubble, shock waves on collapse, and, in pursuit, secondary cavitation [36]. Meire and De Moor highlighted several disadvantages of conventional irrigation and also sonic or ultrasonic solution activation in minimally invasive endodontics, such as tip wedging, limited irrigant penetration, and the vapor lock effect. Because lasers overcome these disadvantages by action mechanism, they concluded that laser-assisted irrigation is superior to conventional irrigation, and ultrasonic as well, and there is a clear trend of advanced antimicrobial effect [36]. The findings of the current study also showed a result consistent with this evidence.

There are several limitations of this study. First of all, in in vitro conditions, it is hard to standardize the remaining necrotic tissue in the root canal lumen because there is no way to diagnose when the tooth has undergone necrosis. However, even if it is a limitation itself, this is an actual clinical situation that clinicians commonly face in daily practice. Therefore, this parameter was selected to reflect the clinical scenario. Additionally, although the preference for 2D radiography for imaging may seem like a limitation, it provides a sufficient and literature-accepted characterization for oval and single-rooted teeth when taken from two directions because the calculation technique is performed in the same way in periapical radiography, CBCT, and micro-CT [2,3,22,23]. As another limitation, although most possible parameters have been standardized because different working principles and methods are used, standardization itself is a significant limitation, as there is a lack of evidence in the literature regarding which tool performs the highest possible level of debridement under which conditions [36]. Finally, endodontics is a field where experience is developed over time with a learning curve. Operator-induced variations may affect the results, especially in the mechanical steps like access cavity preparation and instrumentation phase. Therefore, all preparations were performed by a blind, well-trained endodontist before group allocation. Another limitation was the difficulty in differentiating the timing of pulp necrosis and, consequently, the amount of necrotic tissue that would be standardized and analyzed. Considering the clinical conditions, this limitation is also controversial regarding evaluation in vivo for patients’ necrotic teeth pulp tissue when conducting root canal treatment and determining the treatment action for this variation. Therefore, this situation may be considered as a reflection of clinical practice and a clinical variation. In this context, to discuss the results of the study statistically, the sample size was determined by performing a power analysis in order to conclude within clinical standardizations and variations.

These findings suggest that incorporating advanced irrigation activation systems such as multisonic ultracleaning by GentleWave, Er:YAG laser by SWEEPS, and Er,Cr:YSGG laser by Waterlase iPlus in endodontic treatments could improve long-term success, even for conservative preparation, contributing to the structural integrity of treated teeth. This research fills the gap for the comparative effectivity of current advanced and conventional techniques and pioneers improving irrigation activation systems for root canal treatment. However, the authors believe that none of the in vitro laboratory studies are able to generalize the results, so even if these results give promising data, but in their own condition, their limitations should be taken into consideration. These future perspectives aim to build on the promising findings of the study and facilitate the translation of advanced protocols into routine clinical practice, ultimately improving patient outcomes in endodontics.

## 5. Conclusions

In necrotic oval-shaped canals with minimally invasive instrumentation, GentleWave (with updated software) was considerably more effective at removing remnants in the apical level debridement. The Er:YAG laser (SWEEPS) and Er,Cr:YSGG laser (Waterlase iPlus) were highly promising, with results close to those of multisonic ultracleaning (GentleWave with updated software). It should be considered that conventional irrigation, and even passive ultrasonic activation, may not be able to provide competent debridement in treating such types of root canals.

## Figures and Tables

**Table 1:** Comparison of remaining area mean values of cross-sectional slides at 1–3 mm apical level.

Groups	N	n	Mean ± SD
Needle irrigation	14	3	15.64 ± 7.23
Multisonic ultracleaning	14	3	1.54 ± 1.46 ^a^
Er,Cr:YSGG laser	14	3	5.04 ± 3.63 ^ab^
Er:YAG laser	14	3	3.54 ± 2.81 ^a^
Passive ultrasonic	14	3	8.40 ± 5.92 ^abcd^

Statistical significance. N—sample size per group; n—number of slides evaluated per sample. Different letter in superscript indicate the difference between groups (^a^—needle irrigation; ^b^—multisonic ultracleaning; ^c^—Er,Cr:YSGG laser; ^d^—Er:YAG laser).

**Table 2:** Means, standard deviations, and statistical significance between each group.

Groups	Mean	SD	1	2	3	4	5
1. Needle irrigation	15.64	7.23	-				
2. Multisonic ultracleaning	1.54	1.46	<0.001 ***	-			
3. Er,Cr:YSGG laser	5.04	3.63	<0.001 ***	0.007 **	-		
4. Er:YAG laser	3.54	2.81	<0.001 ***	0.298 ^ns^	0.599 ^ns^	-	
5. Passive ultrasonic	8.4	5.92	<0.001 ***	<0.001 ***	0.011 *	<0.001 ***	-

Note. * *p* < 0.05, ** *p* < 0.01, *** *p* < 0.001, ^ns^ no significance.

## Data Availability

The data related to this study’s findings are available from the corresponding author upon reasonable request.

## Abbreviations

The following abbreviations are used in this manuscript:

NaOCl	Sodium hypochlorite
EDTA	Ethylenediaminetetraacetic acid
NI	Needle irrigation
PUI	Passive ultrasonic activation
wt	Weight
vol	Volume
M	Mean
SD	Standard deviation
